# Sociocultural factors influencing breastfeeding practices in two slums in Nairobi, Kenya

**DOI:** 10.1186/s13006-016-0092-7

**Published:** 2017-01-11

**Authors:** Milka Wanjohi, Paula Griffiths, Frederick Wekesah, Peter Muriuki, Nelson Muhia, Rachel N. Musoke, Hillary N. Fouts, Nyovani J. Madise, Elizabeth W. Kimani-Murage

**Affiliations:** 1 African Population and Health Research Center (APHRC), Nairobi, Kenya; 2 Centers for Global Health and Human Development; Loughborough University, Loughborough, UK; 3 MRC/Wits Developmental Pathways for Health Research Unit, Faculty of Health Sciences, University of the Witwatersrand, Johannesburg, South Africa; 4 Departments of Pediatrics, University of Nairobi, Nairobi, Kenya; 5 Department of Child and Family Studies, University of Tennessee, Knoxville, USA; 6 Center for Global Health, Population, Poverty, and Policy University of Southampton, Southampton, UK

**Keywords:** Culture, Sociocultural, Breastfeeding, Slums, Kenya

## Abstract

**Background:**

Despite numerous interventions promoting optimal breastfeeding practices in Kenya, pockets of suboptimal breastfeeding practices are documented in Kenya’s urban slums. This paper describes cultural and social beliefs and practices that influence breastfeeding in two urban slums in Nairobi, Kenya.

**Methods:**

Qualitative data were collected in Korogocho and Viwandani slums through 10 focus group discussions and 19 in-depth interviews with pregnant, breastfeeding women and community health volunteers and 11 key-informant interviews with community leaders. Interviews were audiotaped, transcribed verbatim, coded in NVIVO and analyzed thematically.

**Results:**

Social and cultural beliefs and practices that result to suboptimal breastfeeding practices were highlighted including; considering colostrum as ‘dirty’ or ‘curdled milk’, a curse ‘bad omen’ associated with breastfeeding while engaging in extra marital affairs, a fear of the ‘evil eye’ (malevolent glare which is believed to be a curse associated with witchcraft) when breastfeeding in public and breastfeeding being associated with sagging breasts. Positive social and cultural beliefs were also identified including the association of breast milk with intellectual development and good child health. The beliefs and practices were learnt mainly from spouses, close relatives and peers.

**Conclusion:**

Interventions promoting behavior change with regards to breastfeeding should focus on dispelling the beliefs and practices that result to suboptimal breastfeeding practices and to build on the positive ones, while involving spouses and other family members as they are important sources of information on breastfeeding.

**Trial registration:**

ISRCTN83692672: December 2013 (retrospectively registered)

## Background

Optimal breastfeeding has been described as one of most effective interventions in reducing infant and child mortality globally [1]. Recent evidence indicates that breastfeeding could save over eight hundred thousand children’s lives and about two hundred mothers’ lives annually [2]. Further, breastfeeding has been associated with increased intelligence, education attainment at adulthood, productivity, earning ability and social development [3, 4]. Conversely, suboptimal breastfeeding is the major cause of over 30% of child deaths, especially in low income settings [5, 6] and is associated with national gross economic losses [7].

Optimal breastfeeding as recommended by the World Health Organization (WHO) includes immediate initiation of breastfeeding, exclusive breastfeeding for six months and continued breastfeeding for at least two years with optimal complementary feeding from six months [8]. Despite the established benefits of optimal breastfeeding, about 60% of infants in developing countries are not exclusively breastfed for six months [9]. Kenya however, has made remarkable progress in exclusive breastfeeding. The number of children exclusively breastfed for six months in the country has doubled over the last five years, from 31% in 2008 to 61% in 2015 [10], marking it as one of the few countries on track to achieving the World Health Organization’s Global Nutrition Target for exclusive breastfeeding [11]. In spite of this progress, four out of ten children are still not exclusively breastfed and about 15% are started on other foods earlier than six months [12]. The situation in the urban slums where about 70% of the urban population resides [13] is alarming, as only about two percent of infants are exclusively breastfed for six months while the mean age of introducing complementary feeding is one month [14]. Cultural beliefs, myths and misconceptions have been highlighted as some of the challenges to optimal breastfeeding in the country [15] and as one of the probable causes of poor breastfeeding practices in the urban slums [14, 16, 17].

Cultural beliefs and norms have a powerful influence on human nutrition [18, 19] and have been identified as among the determinants of breastfeeding practices [7]. Several studies have also emphasized the need to understand and incorporate cultural beliefs and practices in design and implementation of health and nutrition interventions [19–21]. The Global Strategy for Infant and Young Child Feeding further emphasizes on the need for those involved in promoting breastfeeding to understand the sociocultural and environmental circumstances around breastfeeding [8].

Although myths, misconceptions and cultural beliefs have been highlighted as among the hurdles to optimal breastfeeding and infant feeding in Kenya, there is a dearth of evidence on the specific cultural beliefs and practices on breastfeeding, especially in the urban slums where poor breastfeeding practices are rampant. This study documents the social and cultural beliefs, practices and misconceptions around breastfeeding in urban slums. Evidence generated can inform design and implementation of interventions and policies to improve breastfeeding and consequently child health and nutrition in these and similar settings.

## Methods

### Study area

The study was conducted in two slums, Korogocho and Viwandani in Nairobi, Kenya where the African Population and Health Research Center (APHRC) has run the Nairobi Urban Health and Demographic Surveillance System (NUHDSS) since 2003. The two slums are about seven kilometers apart covering a total area of approximately one Km^2^ with about 70,000 individuals from 28,500 households. They are characterized by poor access to health and education facilities, food insecurity, poor health and nutrition indicators and high early pregnancy rates [22–25]. The study area has over 15 ethnic groups with the majority being the Kikuyu, Luhya, Kamba and Luo [26]. Although each ethnic group speaks a specific ethnic language, the commonly used language is Swahili, the national language spoken across all the ethnic groups.

### Recruitment and training of field staff

Ten field interviewers, all university graduates and with prior experience in qualitative data collection were recruited and trained for five days. The training content included the study objectives, qualitative data collection techniques, the interview guides and research ethics. The field interviewers were also involved in the pretesting and refining of the interview guides in the field [27]. Pilot interviews and debriefing sessions were conducted during the training. Some of the researchers accompanied the field team in pilot interviews and participated in the debriefing sessions.

### Recruitment of study participants and data collection

Data were collected in April 2012 through 10 Focus Group Discussions (FGDs), 11 Key Informant Interviews (KIIs) and 19 In-Depth Interviews (IDIs) in Korogocho and Viwandani. A total of 110 participants; 20 men and 90 women were recruited through purposive sampling depending on the category of respondents, taking into account different ethnicity, religious affiliation and village of residence. To be eligible for the study, the participants had to be residents of the study areas and be willing to voluntarily participate in the study. Characteristics of respondents and the interview details are given in Tables 1 and 2 respectively.

**Table 1:** Interviews by type and category

Interviews/study site	Korogocho	Viwandani	Total
By type of interview (*n* = interviews)			
In-Depth Interviews	11	8	19
Focus Group Discussions	6	4	10
Key Informant Interviews	6	5	11
By Category (*n* = interviews)			
*Focus Group Discussions*			
Village Elders	1	1	2
Young Mothers (below 25 years)	2	1	3
Older mothers (25+ years)	2	1	3
Community Health Workers (CHWs)	1	1	2
*In-Depth Interviews*			
Pregnant Mothers	2	1	3
Breastfeeding mothers	5	4	9
Mothers of children aged under 5 years (Not breastfeeding)	2	1	3
HIV positive mothers	2	2	4
*Key Informant Interviews*			
Healthcare Workers	1	1	2
Religious Leaders	1	1	2
Traditional Birth Attendants	1	1	2
Women Leaders	2	1	3
Youth Leaders	1	1	2

**Table 2:** Sociodemographic characteristics of the study participants

Sociodemographic characteristics (*n* = individuals)	Men	Women	Total
Age (Years)			
Mean age	41.8	29.3	31.58
< 25 years	1	39	40
> = 25 years	19	51	70
Religion			
Christian	19	74	93
Muslim	1	14	15
Missing	0	2	2
Ethnic background			
Kikuyu	2	25	27
Kamba	5	17	22
Luo	4	23	27
Luhya	4	9	13
Somali	0	4	4
Other	5	12	17
Education status			
None	0	7	7
Preprimary (Early Child Development)	4	11	15
Primary	4	50	54
Secondary	7	18	25
Post-Secondary/College	5	4	9
Occupation			
Casual worker	0	11	11
Community Health Worker (CHW)	1	9	10
Health Worker (Nurse, Clinical Officer)	1	2	3
Social Worker/Religious Leader	3	0	3
Self-employed/business including artisans	11	27	38
Community leader	4	0	4
Not working (including housewife, student)	0	37	37
Missing	0	4	4
Marital status			
Married	18	50	68
Widowed	0	4	4
Not married/Single/Separated	2	34	36
Missing	0	2	2
Slum			
Korogocho	8	59	67
Viwandani	12	31	43

FGDs were conducted with village elders (2), young mothers (3), older mothers (3) and community health workers (2) in order to gain a deeper understanding of the local context, norms and cultural practices influencing breastfeeding and child feeding practices in the two slums.

KIIs were conducted with individuals who had lived or worked and interacted with the study communities and had a good understanding of the communities’ breastfeeding and infant feeding practices. These included; women group leaders (3), youth group leaders (2), traditional birth attendants (2), religious leaders (2) and healthcare workers (2). The aim was to gain an overview of the attitudes and practices regarding breastfeeding and infant feeding practices in the community.

IDIs were conducted with pregnant women (3), breastfeeding mothers (9), non- breastfeeding mothers (3) and HIV positive mothers (4) with an aim of understanding the knowledge, attitudes and practices on breastfeeding, specific to these groups.

The interview guides were composed of open ended questions and addressed the following issues; nutritional status of children in the community, knowledge, attitudes and practices around breastfeeding and infant feeding, cultural beliefs and practices around breastfeeding and infant feeding as well as challenges and enabling factors to optimal breastfeeding and infant feeding practices. The questions were derived from the study objectives and issues emerging from previous interviews that required further interrogation.

Interviews were conducted in *Swahili*, the commonly used language in the study area. Each interview was conducted by a moderator and a note taker, audiotaped and transcribed verbatim. Concurrent transcription and translation was done by two graduates with experience in transcription, who had participated in the training of the interviewers and the pilot sessions.

### Data analysis

Transcribed word files were transferred to NVIVO 10 software (QSR International Pty Ltd, Don Caster, Victoria, Australia) for coding. Coding and interpretation was done by two members of the research team to ensure comprehensiveness and reliability in application of the coding process [28]. Final checks for consistency of the application of the codes were undertaken with a third member of the research team. Codes were based on main themes derived from literature and the study objectives. Analysis of the transcripts was mainly deductive, based on preexisting themes identified from existing literature [28]. Peer debriefing was also conducted during the data collection process to discuss other emerging themes. These themes were investigated further in the subsequent interviews and considered in the analysis, and included breastfeeding practices among young mothers and the sex differences in infant feeding practices.

## Results

**Fig. 1:**
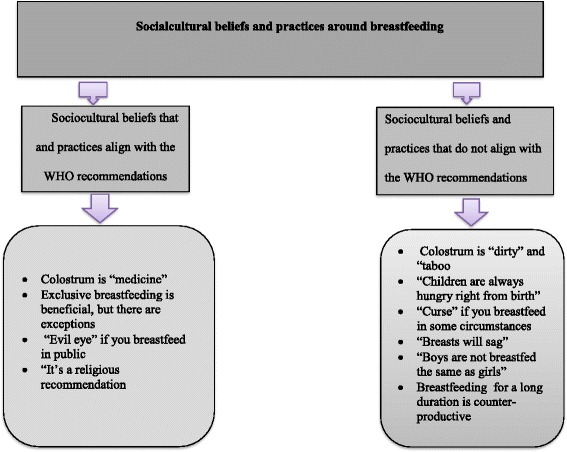
Themes and sub themes on social and cultural beliefs and practices around breastfeeding

Various social and cultural beliefs and practices related to breastfeeding in the community were identified from the narratives of the study participants. Some of the beliefs align with the WHO recommendations while others do not align with the WHO recommendations. The results are organized into eight subthemes, as summarized in Fig. 1. Most of the participants reported to have learnt or observed these beliefs and practices mainly from parents, grandparents, spouses or older women in the community.

### Colostrum is “medicine” but ‘taboo’ in some communities

Views on feeding colostrum to babies varied among the participants. The majority had the opinion that it is highly nutritious *‘it has a balanced diet’,* medicinal and that it confers immunity to the newborn against some diseases. Others however described it as dirty due to its color and consistency *‘thick and yellowish’* or ‘*watery*’ which differs from ‘*normal*’ milk.

Among the *Sukuma* (ethnic group in Korogocho, believed originate from Tanzania)*,* feeding the baby on colostrum is discouraged and regarded as taboo; “*Sukuma people from Tanzania called ‘Mapenga’, do not breastfeed on colostrum, even if the mother doesn’t have fingers she would rather ask for assistance to express colostrum for the first 2 days and throw away” (FGD, village elders- Korogocho).*

### “Children are always hungry, right from birth”

Prelacteal feeding in this population is mainly practiced due to the perception that the baby is born naturally hungry, requiring other foods before breast milk starts flowing. Inadequate milk in the first few days and crying were also identified as reasons for prelacteal feeding. *“2 or 3 day after birth, if the child cries, they will boil water and put some salt and sugar, to give the child to stop its stomach ache” (FGD CHWs- Viwandani).*

The practice was mostly reported among the *luhya* ethnic group. The most common prelacteal feeds were plain water, a sugar or salt solution, glucose, cow’s milk or light porridge. *“Luhya children are always hungry right from birth so you give it. So even if the baby doesn’t have milk, you will be forced to prepare some light porridge and give it” (FGD, CHWs- Korogocho*).

### Exclusive breastfeeding is beneficial, but there are exceptions

There is wide knowledge on the benefits of exclusive breastfeeding and the association it has with physical and intellectual development. Babies who are exclusively breastfed were said to be very intelligent, strong, with better health compared to those who are not. A young mother for instance believed that children who are exclusively breastfed perform well and go to the best schools in the country *“If breastfed for six months they become very clever (whistles) Maranda high school…First class” (FGD, young mothers- Korogocho).*

Despite the knowledge on the benefits of exclusive breastfeeding however, perceptions that hinder mothers from exclusively breastfeeding their children were noted. These include the perception that some mothers do not have adequate breast milk to practice exclusive breastfeeding for six months, that breastfeeding exclusively or for six months causes difficulties in initiating complementary foods; “*Some children are used to breast milk that they refuse other foods at 6 months’… to make them eat you have to stop breastfeeding” (FGD with older mothers- Viwandani).* Also that breast milk alone was insufficient to support optimal growth of babies perceived to be either too small to too big, *“Some children are born heavy in terms of weight…and they don’t get full on breast milk. You give other foods like porridge even before one month.” (FGD, older mothers- Korogocho).*

### “Bad Omen (Curse)” if you breastfeed in some circumstances

It is strongly believed in some ethnic groups, especially the Luo and Luhya ethnic groups, that a mother’s milk becomes unclean if she is involved in extramarital relationships with men who are not the baby’s father. It is considered a bad omen or curse ‘chira’ if the mother continues to breastfed while engaging in such relations, which could lead to death of the baby. As a result some mothers cannot continue breastfeeding if they engage in these relations unless some cleansing ritual is done. This however does not directly associate breastfeeding with marital faithfulness or non- breastfeeding with promiscuity in the general community *“The more you have sex with many people the higher the chances the child will die when you are breastfeeding…It is a taboo for a mother who is breastfeeding to have sex with different men” (FGD, CHWs – Viwandani).*

Further, breastfeeding women have to undergo cultural cleansing rituals ‘manyasi’ after having confrontations with community members or their spouse before they are allowed to resume breastfeeding. If not done, discontinuation of breastfeeding is recommended. This is common among the Luo and Luhya ethnic groups. *“For Luo and Luhya, if I am breastfeeding and I quarrel with my neighbor or husband I can’t just come home and breastfeed, I have to be given herbs ‘manyasi’ first or I completely stop to breastfeed” (FGD, older mothers- Korogocho).*

### “Evil eye” if you breastfeed in public

Some women fear breastfeeding in public as they could be watched by people in the community who are believed to have an ‘evil eye’ (malevolent glare which is believed to be a curse associated with witchcraft). They fear that if a person with an “evil eye” looks at them while breastfeeding, breast milk will dry up or the mother will develop breast ‘sores’. This may prompt some mothers to avoid breastfeeding or start bottle-feeding, especially when attending public gatherings, or generally being in public.

*“In this community we have people who have an evil eye and they are known that if so and so sees you breastfeed, milk will dry and so mothers will breastfeed in a certain style when they are outside the house…Even in the clinic, some mothers do not want people to see them breastfeed; they claim they will bewitch them” (FGD, CHWs- Viwandani.)*

### “Breasts will sag”

A common belief among young mothers in the community is that breastfeeding for a long duration will make their breasts sag and render them unattractive. *“For some, the more they breastfeed, the more they lose weight…or the more they breastfeed the baby the more the breasts sag/flatten (laughter)…so they stop breastfeeding the baby” (FGD, young mothers -Korogocho).* For this reason young mothers reported stopping breastfeeding their children as early as possible to prevent their breasts from sagging. *“If you are a girl, you don’t want your breasts to sag, so you don’t breastfeed…(FGD, young mothers -Viwandani).*

### “It’s a religious recommendation”

Islam was said to recommend breastfeeding for an exact number of years which is equated to sharing one’s wealth with their children. This issue emerged from discussions with mothers, and was further investigated by interviewing Muslim religious leaders in the community. For this reason, mothers from this religious group reported to endeavor to breastfeed their children for at least two years. “*Muslim we are supposed to breastfeed for two years and if you don’t breastfeed your baby for those two years, as our book says…you have not given your child half your wealth. You should not exceed the two years even by one day” (IDI, young mother-Korogocho)*. However, the Quran seems more lenient on the breastfeeding recommendation than how people presented the case, as the decision about breastfeeding and the time of weaning is expected to be a mutual decision by both parents, in consideration of what is best for their family as depicted by the Quran verse;

*“Mothers shall breastfeed their children for two whole years…for those who wish to complete the term. If they both (parents) decide on weaning, by mutual consent, and after due consultation, there is no blame on them” (2:233).*

### “Boys are not breastfed the same as girls”

The duration of breastfeeding is said to be shorter in boys than girls in some communities, due to a common belief that boys breastfeed ‘a lot’ and ‘weaken’ the mother. *“Mothers with male children complain that the children really breastfeed often, so they cannot be allowed to breastfeed for two years but are allowed for maybe a year and a half or less. The female girl is advantaged because they may breastfeed longer up to maybe three years, a male child may even make the mother feel dizzy after suckling” (FGD with older mothers- Korogocho).* As a consequence there is a tendency of earlier introduction of other foods and shorter breastfeeding duration in boys than girls *“Boys breastfeed a lot (laughter) so they are stopped when still young” (FGD, older mothers- Viwandani).*

## Discussion

This study provides a breadth of knowledge about the social and cultural beliefs and practices that influence breastfeeding practices in Korogocho and Viwandani slums in Nairobi. Some of the beliefs align with the WHO recommendations on breastfeeding and positively influence the translation of the recommendations into practice, these include the common belief that colostrum is natural medicine and that breast milk promotes brain and intellectual development. These positive beliefs could be used in behavior change interventions to develop messages on optimal breastfeeding, as presented in a study by Samega-Janneh et al. where the use of local practices that align with the recommended infant feeding practices were successfully used to develop messages on optimal child feeding and care practices [21].

Emphasis on the need for prelacteal feeding was said to be common among ethnic groups from the western region of the country and may hamper immediate initiation of breastfeeding after birth as recommended [8]. According to the Kenyan Demographic and Health Survey (2010), the western region of Kenya had the highest prevalence of prelacteal feeding (68%) and the lowest prevalence of immediate initiation of breastfeeding (34%) compared to other regions in the country [29]. Cultural influences with prelacteal feeding has also been documented in other African countries [30, 31] and is a major cause for delayed initiation of breastfeeding, and consequent increased risks of neonatal infections and death [32]. It is also associated with unsuccessful exclusive and all breastfeeding [33]. Community based interventions directed towards promotion of optimal maternal and infant nutrition should dispel the practice of prelacteal feeding.

The beliefs that breast milk alone is not enough to support optimal growth and that some mothers naturally do not produce enough milk, were common reasons for not practicing exclusive breastfeeding and for the introduction of other foods earlier than six months. Mothers who were perceived to have inadequate milk were also likely to stop breastfeeding their children earlier than the recommended two years. This concurs with a quantitative study on ethnicity and breastfeeding in Nairobi, Kenya by Watson et al., which documents culture as a major predictor of breastfeeding duration [17]. Studies in Africa and other parts of the world, also indicate that perceptions that breast milk alone is inadequate and anxiety over breast milk insufficiency as the major reasons for early cessation of breastfeeding [34–37].

Among young and teenage mothers, the belief that breastfeeding causes breasts to ‘sag’ was very common. Despite the existing evidence by Rinker et al. that breastfeeding has no effect on breast aesthetics [38], this perception was reported to be a major reason for early discontinuation of breastfeeding or lack of breastfeeding. Perceptions that breastfeeding interferes with breast aesthetics has been indicated as a challenge to exclusive breastfeeding in other settings [16, 34]. This is a major concern especially with the current statistics on increasing teenage pregnancies in slum communities in Kenya [23], accentuating the need for youth friendly interventions and programs to promote optimal breastfeeding and infant feeding among young mothers.

Sex differences in breastfeeding were also apparent in this study. The main reason for these differences, as reported by participants, is the belief that boys are not adequately satisfied by breast milk alone and that their breastfeeding demands are higher than most mothers can sustain for two years or beyond. The Kenyan Demographic and Health Survey, 2010 and a study in urban slums also indicate greater likelihood of prelacteal feeding in boys (45%) than in girls (39%) and a slightly longer duration of breastfeeding in girls as compared to boys [14]. Reassuring mothers that breast milk remains an important part of an infant’s diet for two years for both girls and boys would be an important intervention message in this context.

The cultural beliefs documented in this study were reported especially by young mothers to have mainly been learnt from parents, grandparents, spouses or from older women in the community. A study in Kenya by Walingo et al. in 2014, indicates that a mother’s partner and close family members may influence her breastfeeding behavior, this has also been evidenced in other settings in Africa [34]. The need for community involvement in nutrition interventions to promote optimal breastfeeding is therefore recommended.

## Conclusions

This study indicates that in urban slum settings in Nairobi, cultural and social beliefs influence breastfeeding practices. The Global Strategy for Infant and Young Child Feeding emphasizes on the need for those involved in promoting breastfeeding to understand the sociocultural and environmental circumstances in breastfeeding, the evidence presented in this study is therefore useful in design and implementation of behavior change interventions targeting improved breastfeeding practices, especially among the urban poor in Nairobi. The messages also need to be tailored to the specific ethnic groups to maximize the chances of success in optimal breastfeeding practices and improved infant nutrition outcomes. To this end, further investigation of the role of ethnicity in this setting is recommended.

## Abbreviations

APHRC: African population and health research center
CHW: Community health worker
FGD: Focus group discussion
IDI: In-depth interview
KII: Key informant interview
NUHDSS: Nairobi Urban health and demographic surveillance system
WHO: World health organization
